# CD16+ Monocytes and Skewed Macrophage Polarization toward M2 Type Hallmark Heart Transplant Acute Cellular Rejection

**DOI:** 10.3389/fimmu.2017.00346

**Published:** 2017-03-24

**Authors:** Thierry P. P. van den Bosch, Kadir Caliskan, Marina D. Kraaij, Alina A. Constantinescu, Olivier C. Manintveld, Pieter J. M. Leenen, Jan H. von der Thüsen, Marian C. Clahsen-van Groningen, Carla C. Baan, Ajda T. Rowshani

**Affiliations:** ^1^Department of Internal Medicine and Transplantation, Erasmus University Medical Center, Rotterdam, Netherlands; ^2^Department of Cardiology, Erasmus University Medical Center, Rotterdam, Netherlands; ^3^Department of Immunology, Erasmus University Medical Center, Rotterdam, Netherlands; ^4^Department of Pathology, Erasmus University Medical Center, Rotterdam, Netherlands

**Keywords:** heart, transplantation, macrophages, monocytes, rejection

## Abstract

**Background:**

During acute heart transplant rejection, infiltration of lymphocytes and monocytes is followed by endothelial injury and eventually myocardial fibrosis. To date, no information is available on monocyte–macrophage-related cellular shifts and their polarization status during rejection. Here, we aimed to define and correlate monocyte–macrophage endomyocardial tissue profiles obtained at rejection and time points prior to rejection, with corresponding serial blood samples in 25 heart transplant recipients experiencing acute cellular rejection. Additionally, 33 healthy individuals served as control.

**Materials and methods:**

Using histology, immunohistochemistry, confocal laser scan microscopy, and digital imaging expression of CD14, CD16, CD56, CD68, CD80, and CD163 were explored to define monocyte and macrophage tissue profiles during rejection. Fibrosis was investigated using Sirius Red stainings of rejection, non-rejection, and 1-year biopsies. Expression of co-stimulatory and migration-related molecules on circulating monocytes, and production potential for pro- and anti-inflammatory cytokines were studied using flow cytometry.

**Results:**

At tissue level, striking CD16+ monocyte infiltration was observed during rejection (*p* < 0.001). Significantly more CD68+CD163+ M2 macrophages were documented during rejection compared to barely present CD68+CD80+ M1 macrophages. Rejection was associated with severe fibrosis in 1-year biopsies (*p* < 0.001). Irrespective of rejection status, decreased frequencies of circulating CD16+ monocytes were found in patients compared to healthy individuals. Rejection was reflected by significantly increased CD54 and HLA-DR expression on CD16+ monocytes with retained cytokine production potential.

**Conclusion:**

CD16+ monocytes and M2 macrophages hallmark the correlates of heart transplant acute cellular rejection on tissue level and seem to be associated with fibrosis in the long term.

## Introduction

During acute cellular heart transplant rejection, infiltration of lymphocytes and monocytes is followed by endothelial injury, structural tissue damage, and eventually myocardial fibrosis (1). Not only T-cells but also monocyte-/macrophage lineage cells are abundantly present in the rejecting heart (2, 3). To date, the rejection-related monocyte–macrophage subset profiles in both tissue and blood compartment are not yet defined in heart transplant recipients. It is also unclear whether rejection-related changes of monocyte–macrophage subsets on tissue level are reflected in circulation.

Expression of CD14 (LPS co-receptor) and CD16 (Fcγ receptor III) define three phenotypically and functionally distinct human monocyte subsets: CD14++CD16− (classical), CD14++CD16+ (intermediate), and CD14+CD16++ (non-classical) monocytes (4). The CD16+ monocytes are considered pro-inflammatory due to production of pro-inflammatory cytokines, such as TNF-α and IL-1β compared to classical monocytes (4, 5). Higher expression of HLA-DR, CD86, and CD54 (ICAM-1) distinguish CD16+ monocytes from the classical ones.

Monocytes are important in many inflammatory diseases. Coronary artery disease patients have higher numbers of monocytes compared to healthy cohorts (6, 7). Local biphasic monocyte accumulation was observed following acute myocardial infarction (8, 9). The monocytes, located in the infarct border zone during the inflammatory phase after infarction, consisted mainly of CD14+CD16− cells, whereas during the proliferative phase, the monocytes in the infarct core showed comparable percentages of CD14+CD16− and CD14+CD16+. Different macrophage populations are also observed following myocardial infarction with pro-inflammatory cells early on followed by reparative macrophages (9).

Related to the state of activation, macrophages can be functionally grouped into two main classes: the M1 (pro-inflammatory) and the M2 (anti-inflammatory), although an increasing number of different phenotypes with intermediate and contrasting features have been described recently (10). Classically, the M1 macrophages can be induced after stimulation of monocytes with IFN-γ and mainly have phagocytic, anti-microbial, and pro-inflammatory functions (11). On the other hand, M2 macrophages are induced after monocyte-stimulation with modulating factors such as IL-4/IL-13, immune complexes, or glucocorticoids and are phenotypically characterized by expression of CD163, CD206, and/or CD204. These macrophages exert anti-inflammatory functions by production of IL-10 and TGF-β (12). M2 macrophages can also produce matrix metalloproteinases contributing to extra cellular matrix turnover and fibrosis (13).

As different monocyte and macrophage subset phenotypes are functionally different in induction and/or maintenance of inflammation or fibrosis, it is important to investigate their role in relation to heart transplant rejection. This information will eventually help identifying key cell types, molecules, and markers, which can serve as diagnostic biomarkers of rejection, and/or as targets for rejection treatment. Here, we aimed to define and correlate monocyte/macrophage profiles in tissue and circulation using endomyocardial biopsies (EMBs) obtained at rejection and time points prior to rejection, and their corresponding serial blood samples in 25 heart transplant recipients experiencing acute cellular rejection. Next, we wondered whether these cellular shifts were associated with structural graft damage and fibrosis. Additionally, blood profiles of non-rejecting heart transplant recipients were compared with 33 healthy individuals using a cross-sectional approach.

## Materials and Methods

### Patient Characteristics

Twenty-five heart transplant recipients underwent protocol surveillance biopsies within the first year after transplantation at the Erasmus University Medical Center (Rotterdam, The Netherlands). Peripheral blood mononuclear cells (PBMC) were collected serially in time: at a time point that the protocol biopsy showed no rejection, and subsequently at a time point of biopsy-proven rejection (median time ± SEM between both time points: 3 ± 1.4 weeks). Additionally, biopsies obtained at 1-year post-rejection were used for Sirius Red staining (median time ± SEM between rejection and post-rejection time points: 56 ± 12.5 weeks). Of note, no rejection episodes occurred between the rejection time point and 1-year post-rejection. Histopathological features were scored according to 2011 and 2005 International Society for Heart and Lung Transplantation guidelines in order to diagnose acute antibody-mediated rejection and acute cellular rejection and to grade EMBs as non-rejection (0R) or rejection (2R) (14, 15).

All studied endomycardial biospies (*n* = 50) showed acute cellular rejection (2R according to 2005 ISHLT classification system) with no signs of histopathologic and immunopathologic evidence of acute antibody-mediated rejection. All biopsies were C4d negative. Intravascular macrophages and neutrophils as well as signs of endothelial injury such as swelling and denudation with congestion and/or hemorrhage were absent. No serologic evidence of anti-HLA antibodies could be detected using Luminex technique.

This study was performed according to the tenets of the Declaration of Helsinki and approved by the Medical Ethical Committee of the Erasmus MC. All patients signed written informed consent. Table 1 lists the transplantation characteristics and the clinical and immunological features of this cohort. In addition, blood samples were collected from 33 healthy individuals [age: median + range: 51 (25–73); male: 42%] and used as control.

**Table 1:** **Clinical and immunological characteristics of heart transplant recipients**.

Characteristics	Recipients (*n* = 25)
Age [median (year), range]	46 (15–64)
Gender (% male)	64%
Primary disease (number of patients, %)	
Cardiomyopathy	16 (64%)
Ischemic heart disease	9 (36%)
Induction therapy	
Horse-antithymocyte globulin	68%
Rabbit-antithymocyte globulin	32%
Maintenance therapy	
Cyclosporine/prednisone/mycophenolate mofetil	56%
Tacrolimus/prednisone/mycophenolate mofetil	44%
HLA mismatches total (median, range)	
Class I mismatches	3 (2–4)
Class II mismatches	2 (1–2)
Ischemia [median (min), range]	170 (137–250)

All patients were treated with horse or rabbit antithymocyte globulin (hATG or rATG) as induction therapy in combination with maintenance calcineurin inhibition (Prograft^®^ or Neoral^®^), mycophenolate mofetil (Cellcept^®^), and steroids; the dose schedule was adjusted according to the local standard protocol.

### Phenotype, Activation, and Co-Stimulatory Molecule Status of Monocytes

**Figure 1:**
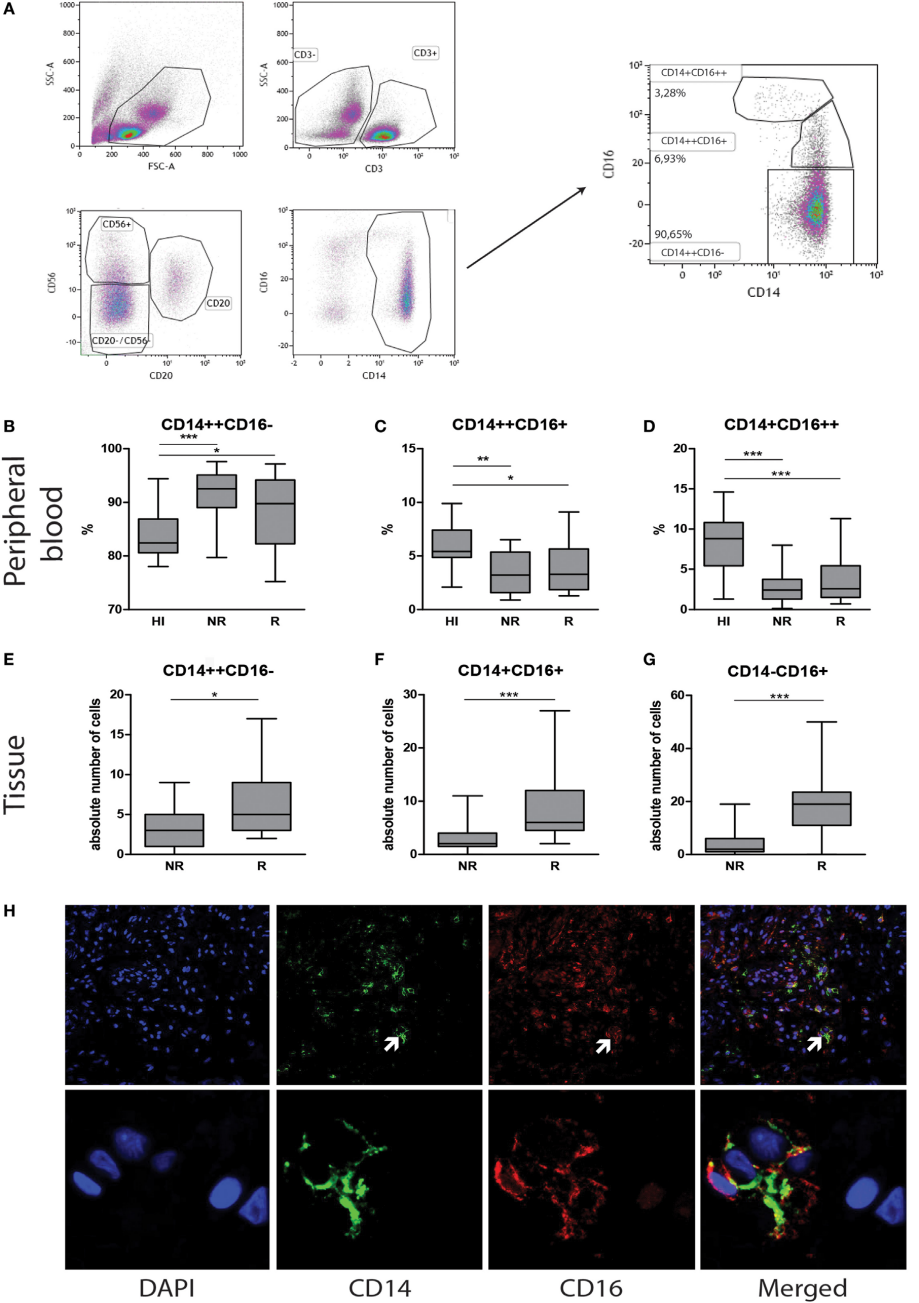
**Contrasting monocyte subsets in blood and tissue during heart transplant rejection**. Representative monocyte subset FACS gating strategy is depicted **(A)**. Blood: the percentage of **(B)** classical CD14++CD16−, **(C)** intermediate CD14++CD16+, and **(D)** non-classical CD14+CD16++ monocytes in healthy individuals (*n* = 33) and heart transplant recipients (*n* = 25) at time points no rejection (NR) and rejection (R) are presented as median ± IQR. Tissue: the absolute numbers of **(E)** CD14+CD16−, **(F)** CD14+CD16+, and **(G)** CD14−CD16+ monocytes in heart transplant biopsies at time points non-rejection (NR; *n* = 25) and R (*n* = 25) are given. A representative overview of double immunofluorescence stainings of rejected endomyocardial tissue is shown; **(H)** CD14 (green) and CD16 (red) at 10× magnification. Detailed co-localization **(H)** of CD14 (green) and CD16 (red) is shown at 63× magnification. Absolute numbers of cells are depicted (*p < 0.05, **p < 0.01, ***p < 0.001).

In order to investigate monocyte phenotype, activation status, and co-stimulatory molecules (HLA-DR and CD54), PBMC were collected from whole blood using Ficol gradient. Labeling and flow cytometric assessment were performed as described before (16, 17). Monocytes were identified based on forward/sideward scatter, lack of expression of CD3, CD20, and CD56, and expression of CD14 and CD16 (Figure 1A).

### Intracellular Cytokine Production

Peripheral blood mononuclear cells were incubated overnight with 10 ng/ml LPS (Sigma-Aldrich) in the presence of GolgiPlug (1:1,000, Becton Dickinson) after pre-stimulation with IFN-γ for 2 h. The cells were then incubated with conjugated primary antibodies in phosphate-buffered saline containing 0.5% bovine serum albumine for 30 min. The antibodies used were CD3-PE, CD14-Pacfic Blue, CD16-PE-Cy7, CD20-PE, and CD56-PE (all Biolegend) at 4°C and were incubated with EDTA for 15 min followed by incubation with FACS permeabilizing solution 2 (BD Biosciences) for 15 min. Next, conjugated antibodies to TNF-α-Percp-Cy5.5, IFN-γ-APC-Cy7, IL-1β-FITC, IL-6-APC, and IL-10-APC and their respective isotype controls (all Biolegend) were added to determine intracellular cytokine production. The cells were washed and analyzed using flow cytometry (FACSCanto II, BD Biosciences) and FACSDiva software (16).

### Immunohistochemical Staining

Immunohistochemistry was performed by an automated staining system (Ventana Benchmark ULTRA, Ventana Medical Systems, USA) using horseradish peroxidase with brown chromogen (3,3′-diaminobenzidine) as enzymatic label. Tissue sections were incubated with antibodies against CD14 (clone 7, mouse-monoclonal IgG2a, 1:100, Leica Biosystems, Newcastle, DE, USA), CD16 (clone sc-20052, mouse-monoclonal IgG1, 1:400, Santa Cruz Biotechnology, Dallas, TX, USA), CD56 (clone 123C3, mouse-monoclonal, Ventana, ready to use, Tucson, AZ, USA), CD68 (clone KP1, mouse-monoclonal, ready to use, DAKO, Carpentaria, CA, USA), CD80 (clone 37711, mouse-monoclonal IgG1, 1:50, R&D systems, Minneapolis, MN, USA), or CD163 (clone EDHu-1, mouse-monoclonal, IgG1, 1:400, AbD Serotec, Raleigh, NC, USA). Two pathologists independently scored these EMBs.

### Immunofluorescence Staining

Tissue sections were incubated with primary mouse monoclonal CD14 IgG2a antibody overnight at 4°C. Secondary goat anti-mouse IgG2a Alexa Fluor 488 (Invitrogen) was applied and incubated for 1 h at room temperature (RT). After washing steps, the second primary antibody mouse-monoclonal CD16 IgG1 was added for 1 h. Next, the secondary goat-anti-mouse IgG1 Alexa Fluor 555 (Invitrogen) was applied for another 1 h at RT. Slides were covered with anti-fading mounting medium containing DAPI (Vectashield, UK) and stored at 4°C until evaluation.

To distinguish CD16 expressing monocytes from the infiltrating CD16+ NK-cells and CD68+ macrophages, CD14/CD16, CD56/CD16, and CD68/CD16 double stainings were performed as described above. Double staining with CD68 (1:1,600, clone KP1, mouse-monoclonal, DAKO, Carpentaria, CA, USA) and CD80 (clone 37711, mouse-monoclonal IgG1, 1:50, R&D systems, Minneapolis, MN, USA) were used to characterize M1-type macrophages. For M2 macrophages, double stainings were performed with CD68 and CD163 (clone EDHu-1, mouse-monoclonal, IgG1, 1:400, AbD Serotec, Raleigh, NC, USA) mAbs. Specific controls are used as displayed in Figure S2 in Supplementary Material.

### Sirius Red Staining

In brief, following deparaffinization slides were rehydrated by passage through decreasing ethanol series, 5 min predifferentation step using 0.2% fosformolybdeen acid followed by 45 min incubation with 0.1% Sirius Red solution. Slides were analyzed using polarization method. Representative pictures were made under polarized light and positive stained area was analyzed by the ImageJ software.

### Image Analyses and Laser Scanning Confocal Microscopy

Endomyocardial biopsy samples were scored using ImageJ IHC analysis software (18). Analyses were performed blinded to the clinical source using scanned Nanozoomer Digital Pathology files. Images of the entire biopsy sample (mean size range: 3.4–3.6 mm) were analyzed at 10× objective magnification.

Confocal microscopy was performed using LSM-700 laser scanning confocal microscope (Carl Zeiss). The entire biopsy samples were scored counting the absolute number of cells as for CD16+CD56−, CD16+CD56+, CD16−CD56+, CD14+CD16−, CD14+CD16+, or CD14−CD16+ using 40× magnification.

### Statistical Analysis

Statistical analysis was performed using Graphpad Prism 6. Statistical significance was evaluated by Mann–Whitney *U* test, *t*-test, and one-way ANOVA. A *p*-value of <0.05 was considered statistically significant.

## Results

### CD16+ Monocytes Are Significantly Decreased and CD16− Monocytes Are Significantly Increased in Peripheral Blood in Heart Transplant Recipients Independent of Rejection Status

We first aimed to investigate how the monocyte subset composition in heart transplant recipients would relate to that of healthy individuals. Although the absolute numbers of monocytes were similar, the percentages of classical CD14++CD16− monocytes [NR: 92% ± 7.5, R: 90% ± 4.5 (median ± IQR)] were significantly higher (*p* < 0.05, *p* < 0.001), and the percentages of intermediate CD14++CD16+ [NR:4% ± 1.5, R:4% ± 3.5 (median ± IQR)] and non-classical CD14+CD16++ monocytes [NR:3% ± 3.5, R:3% ± 3.5 (median ± IQR)] were significantly lower in heart transplant recipients than healthy controls [83% ± 5.25, 5.5% ± 3.5, 9% ± 4.75 (median ± IQR)] (*p* < 0.001) (Figures 1B–D). Moreover, no subset differences could be detected between non-rejection and rejection time points.

Circulating CD3+ T cell frequencies were significantly increased during rejection as compared with non-rejection time points which is consistent with previous findings (19) (*p* < 0.001) (Figure S1 in Supplementary Material).

### CD16+ Monocytes Are Significantly Increased in Rejecting EMBs

Serial EMBs were stained using double immunofluorescence labeling with CD14 and CD16. Co-localization and membranous staining of CD14 and CD16 is shown in rejected tissue (Figure 1H). Absolute numbers of stained cells were counted using 20× magnification field by confocal microscopy. Considering the fact that mean surface area of the total biopsies ranged between 3.4–3.6 mm^2^, there was no need to correct the data for the size of biopsies. Absolute numbers of CD14+CD16− monocytes were significantly higher during rejection compared to non-rejection biopsies (*p* < 0.05). Although usually minor subsets in peripheral blood, both CD14+CD16+ and CD14−CD16+ subsets were prominantly increased during rejection in tissue compared to the prior non-rejection time point (*p* < 0.001, Figures 1E–G).

**Figure 2:**
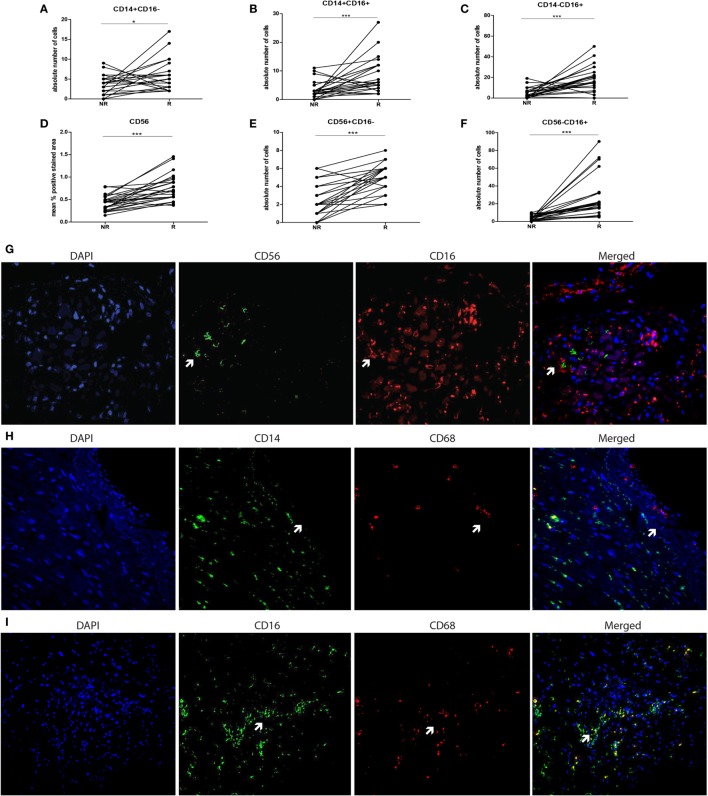
**CD16+ cells in graft tissue are monocytes and accumulate upon rejection**. The expression of CD14 and CD16 was analyzed in biopsies at two time points; non-rejection (NR; *n* = 25) and rejection (R; *n* = 25) and compared intra-individually. The absolute number of cells is depicted for **(A)** CD14+CD16−, **(B)** CD14+CD16+, and **(C)** CD14−CD16+. The expression of **(D)** CD56 was measured using immunohistochemistry, analyzed by ImageJ and depicted as mean % positive stained area. The absolute numbers of cells of **(E,F)** CD56+CD16− and CD56+16+ were determined. A representative overview of rejecting endomyocardial tissue stained for **(G)** CD56 (green) and CD16 (red) at 20× magnification is shown. A representative overview of rejecting endomyocardial tissue stained for **(H)** CD14 (green), **(I)** CD16 (green) and CD68 (red) at 20× magnification is given (*p < 0.05, ***p < 0.001).

To verify whether CD16+ cells were either CD14+ monocyte or CD56+ NK-cell or CD68+ macrophage, double immunofluorescence stainings were performed using CD16/CD56, CD14/CD68, and CD16/CD68 labeling (Figures 2G–I). Individual biopsy analyses showed hardly any co-localization between CD56 and CD16 staining (Figure 2G). Intra-individual analysis of the monocyte subsets showed high numbers of CD16+ expressing cells at rejection (Figures 2A–C). Although the simultaneously present CD56+ NK cells were very low in absolute numbers at rejection compared to non-rejection time points, this difference appeared to be statistically significant between these two time points (Figure 2D). The absolute numbers of infiltrating CD56−CD16+ cells were significantly higher compared to CD56+CD16− within rejecting tissue (*p* < 0.001, Figures 2D–F). Similarly, representative confocal images could hardly show co-localization between CD14- and CD16-positive cells with CD68 surface-expression using CD14/CD68 and CD16/CD68 double stainings of all biopsies (Figures 2H–I). Based on these data, the CD14−CD16+ tissue pool can be considered as monocytes.

### Rejection Is Reflected by Increased Expression of HLA-DR and CD54 by CD16+ Peripheral Blood Monocytes

**Figure 3:**
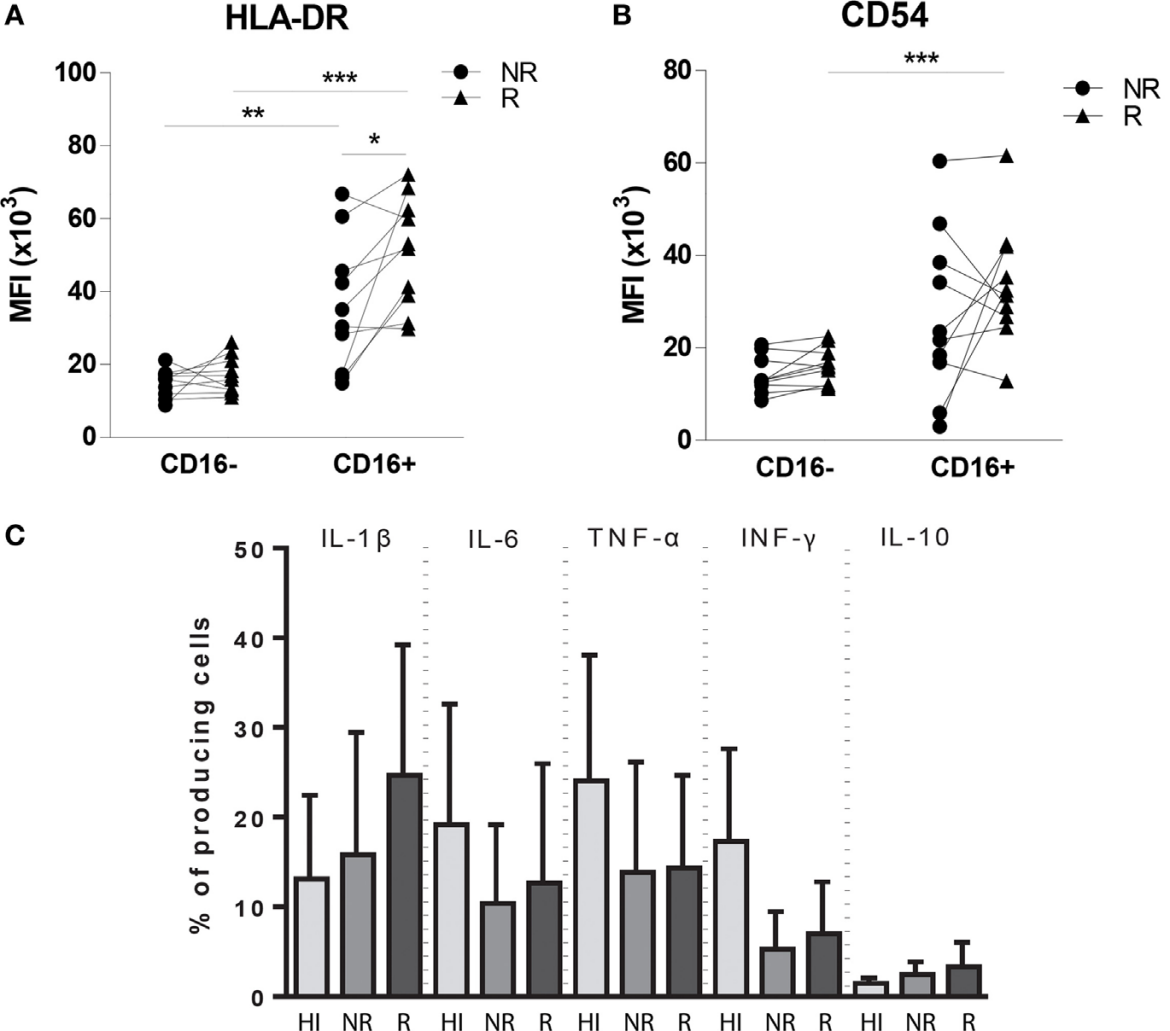
**Phenotypic and functional characteristics of circulating monocytes in heart transplant recipients compared with healthy individuals**. Expression levels of **(A)** HLA-DR, **(B)** CD54 (ICAM-1) are increased in CD16+ monocyte pool during rejection compared to non-rejection. The percentages of IL-1β-, IL-6-, TNF-α-, IFN-γ-, IL-12-, and IL-10-producing monocytes of both healthy individual group (*n* = 9−14) and heart transplant recipients (*n* = 10) are shown at both NR and R time points after LPS stimulation **(C)** (mean ± SEM) (*p < 0.05, **p < 0.01, ***p < 0.001).

To compare monocyte subsets at functional level, expression of co-stimulatory and migration-related molecules by CD16− and CD16+ blood monocyte populations were studied in 10 patients at non-rejection and rejection time points. The HLA-DR expression level was significantly increased on CD16+ monocytes compared with CD16− monocytes at both non-rejection and rejection time points (Figure 3A). During rejection, HLA-DR expression by CD16+ monocytes was even significantly higher as compared to non-rejection time point before. CD16+ monocytes express CD54 at a higher degree compared to CD16− monocytes and this CD54 expression was, although statistically not significant, enhanced during rejection (Figure 3B).

### Pro-inflammatory Cytokine Production Potential of Monocytes in Heart Transplant Recipients Is Preserved and Independent of Rejection Status

To explore the cytokine production capacity of monocytes in 10 heart transplant recipients, we preferred to investigate production of pro-inflammatory cytokines IL-1β, IL-6, TNF-α, and IFN-γ and anti-inflammatory cytokine IL-10 by *in vitro* experiments after LPS stimulation and compared this with healthy individuals (Figure 3C) because localizing cytokine expression on tissue level is generally considered to be associated with a high rate of false positive and false-negative results. The percentage of IFN-γ-producing cells was higher in healthy individuals compared with heart transplant recipients (*p*: 0.112), whereas the production potential of IL-1β, IL-6, TNF-α, and IL-10 was similar between heart transplant recipients and healthy individuals. These findings indicate that, despite the use of potent immunosuppressive drugs in heart transplant recipients, monocytes still remain capable of cytokine production, oftentimes at a similar level as in healthy individuals.

### M2 Macrophages Increase in Rejecting Endomyocardial Tissue Compared to Non-Rejection Time Point

**Figure 4:**
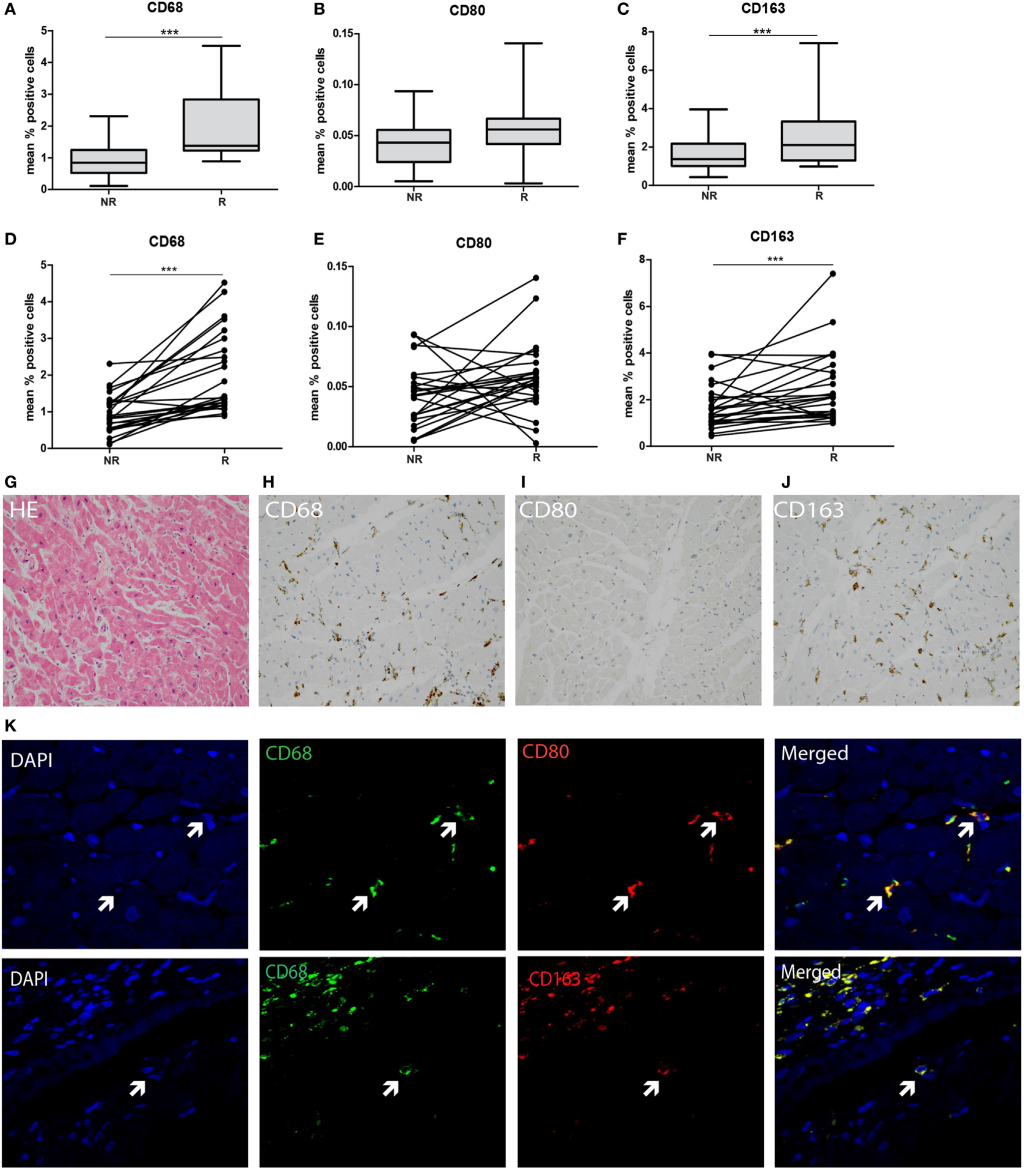
**M2-type macrophages predominate in transplanted endomyocardial tissue and increase upon rejection**. The expression of **(A)** CD68, **(B)** CD80, and **(C)** CD163 was measured using immunohistochemistry, analyzed by ImageJ and depicted as mean % positive stained area. The expression of **(D)** CD68, **(E)** CD80, and **(F)** CD163 was analyzed intra-individually at rejection and non-rejection time points. Representative histological and immunohistochemical images are shown at 20× magnification **(G)** HE, **(H)**, CD68, **(I)** CD80, and **(J)** CD163. Co-localization of **(K)** CD68 and CD80 (M1 macrophage), and CD68 with CD163 (M2 macrophages) is shown at 40× magnification (***p < 0.001).

To explore the type of tissue-infiltrating macrophages, CD68, CD80, and CD163 expression was tested using immunohistochemistry and quantified using ImageJ analysis. A significantly increased presence of CD68+ macrophages was detected in rejected tissue compared to non-rejected tissue in both grouped and intra-individual analysis (*p* < 0.001, Figures 4A,D,G,H). To investigate whether these macrophages are of M1 or M2 origin, adjacent immunohistochemical and double immunofluorescence staining were performed using CD68+CD80 (M1) and CD68+CD163 (M2) combinations. Confocal microscopy was used to show co-localization (Figure 4K). CD80 was hardly expressed by CD68+ macrophages in the endomyocardial tissue (Figures 4B,E,I). Expression increased, albeit not significantly, with rejection, but levels of CD80-expressing cells remained low. In contrast, the vast majority of CD68+ macrophages co-expressed CD163 in biopsies of both rejecting and non-rejecting tissue (Figures 4C,F,J,K) showing a significant increase upon rejection.

### Severe Persistent Fibrosis at Rejection Which Is Irreversible over Time

**Figure 5:**
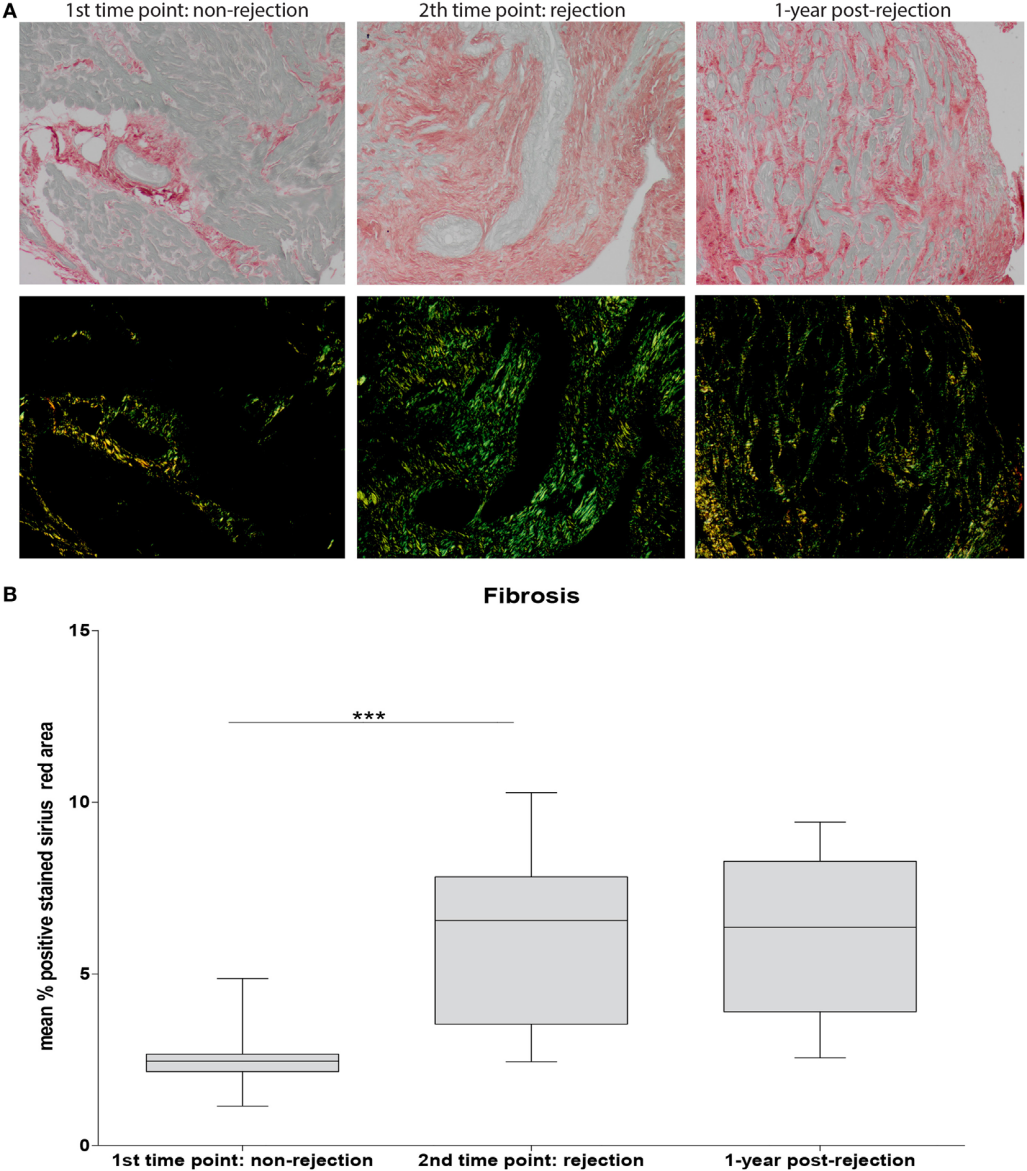
**Severe fibrosis at rejection which is irreversible over time**. Sirius Red staining was used to assess fibrosis. Representative histological images for non-rejection time point (NR; first time point), rejection time point (R; second time point), and 1-year post-rejection time point (1-year post-rejection, third time point) are shown at 20× magnification **(A)**. Fibrosis is quantified as mean % positive stained Sirius Red area **(B)** (mean ± SEM) (***p < 0.001).

To investigate the association between the detected cellular shifts and the degree of fibrosis, the positive Sirius Red stained area at non-rejection, rejection, and approximately 1-year post-rejection was measured. A significantly increased degree of fibrosis was found at rejection compared to non-rejection time point persisting at 1-year post-rejection (*p* < 0.001: Figure 5B). Fibrosis was mainly localized in interstitium and the perivascular areas showing focal collagen accumulation (Figure 5A).

## Discussion

Here, we present an in-depth analysis of peripheral blood and tissue monocyte/macrophage profiles of 25 heart transplant recipients experiencing acute cellular rejection. We found contrasting monocyte subset profile in blood and tissue during rejection with prominent presence of CD16+ monocytes and M2 macrophages at the tissue level. Increased classical monocytes and simultaneously decreased fractions of CD16+ monocytes signify the monocyte subset composition in heart transplant recipients compared to healthy individuals. No numerical differences were noted between rejection and non-rejection conditions. However, rejection was reflected by a significantly increased expression of HLA-DR and CD54 within the circulating CD16+ monocyte pool pointing toward a higher activation grade, antigen presentation potential, and increased migratory capacity of the activated monocytes toward the graft. In line, at tissue level, significantly more CD16+ monocytes, especially of CD14-negative phenotype were detected. Also, significantly more CD68+CD163+ M2 macrophages were documented during rejection. CD68+CD80+ M1 subtype remained a minute subset. The finding of significantly increased fibrosis at rejection, which was also persistently detectable in 1-year biopsies together with the accumulation of CD16+ monocytes and M2 macrophages, indicates an association between these cellular shifts in induction of the prolonged damage to the heart transplant tissue.

On tissue level, we detected significantly higher frequencies of CD16+ monocytes in the rejecting heart tissue. We showed that CD16+ tissue-infiltrating cells are monocytes as hardly co-localization with CD68 and CD56 could be detected ruling out macrophage or NK cell phenotype as the cell source. M2 macrophages accumulate increasingly in tissue during rejection suggesting that the presence of CD16+ monocytes, with a presumed pro-inflammatory nature, and anti-inflammatory IL-10-producing M2 macrophages are parts of a micro-environmental balance within the endomyocardial tissue. Higher tissue macrophage frequencies are known to predict worse graft outcome (20). Future research is needed to investigate monocyte–macrophage profiles during acute antibody-mediated rejection.

**Figure 6:**
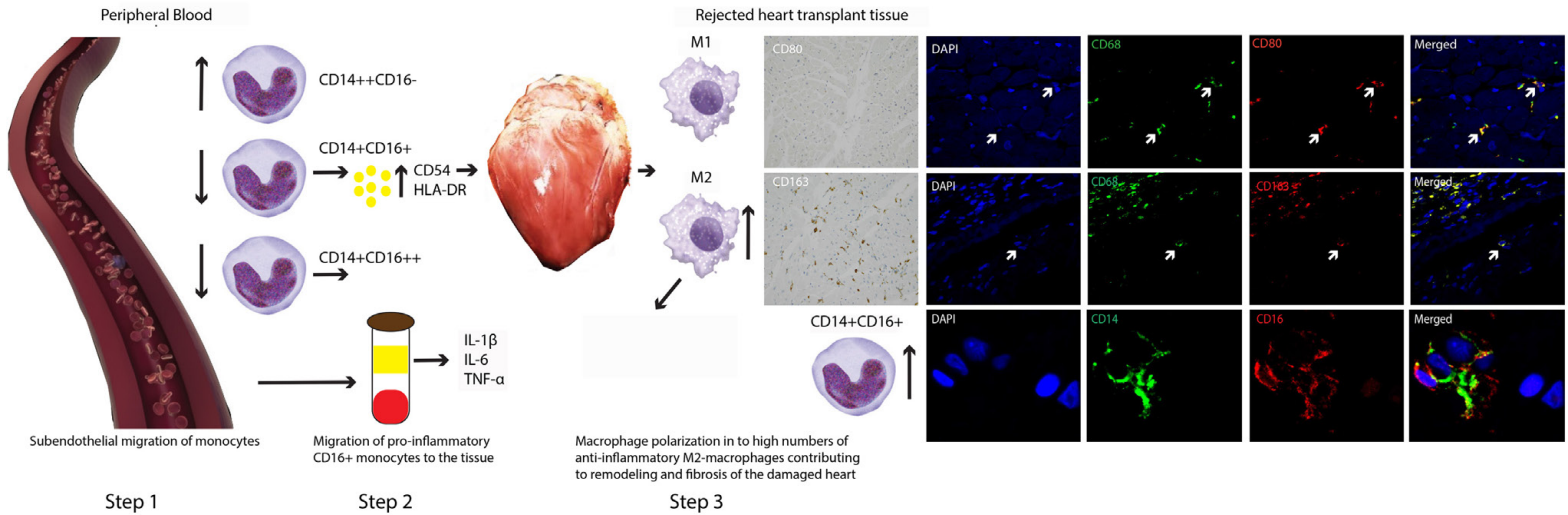
**A model for contrasting findings between blood and tissue monocyte–macrophage lineage cells during heart transplant rejection**. The putative scenario of events based on our findings is described in Section “Discussion.” Briefly, step 1: subendothelial migration of monocytes, step 2: migration of pro-inflammatory CD16+ monocytes toward the tissue, and step 3: macrophage polarization in to high numbers of anti-inflammatory M2-macropghages contributing to remodeling and fibrosis of the damaged heart.

In Figure 6, we attempt to visualize a model based on our data. It is tempting to think that preferentially CD16+ monocytes will leave circulation and enter the graft at transplantation, causing vasculopathy in due time (21). Rejection results in an even higher influx of activated CD16+ monocytes producing pro-inflammatory cytokines. At the same time, a counterbalanced predominance of anti-inflammatory M2 macrophages contributes to the remodeling and fibrosis of the damaged heart tissue (22).

In conclusion, although the numbers of included patients in this explorative study are limited, fibrosis is investigated by Serius Red stainings and not by cardiac MRI with delayed gadolinium pre- and post-rejection, to our knowledge, this is the first report on matched serial blood samples and EMBs at time points prior to rejection and at rejection. Here, we showed that CD16+ monocytes and M2 macrophages hallmark the correlates of acute cellular rejection on tissue level and seem to be associated with fibrosis after heart transplant rejection in the long term. The elucidation of the molecular mechanisms underlying these cellular shifts may lead to discovery of new molecular biomarkers indicating the immunological graft status and may help finding new molecular targets for specific immunotherapy.

## Author Contributions

TB contributed in the process of writing, experimenting, design, discussing, and data analyses. KC contributed in the process of writing, design, discussing, and data analyses. MK contributed in the process of experimenting, design, discussing, and data analyses. AC contributed in the process of writing, sample collecting, and discussing. OM contributed in the process of writing, sample collecting, and discussing. PL contributed in the process of writing and discussing. JT contributed in the process of writing, sample collecting, data analyses, and discussing. MG contributed in the process of writing, sample collecting, data analyses, and discussing. CB contributed in the process of writing and discussing. AR contributed in the process of writing, experimenting, design, discussing, and data analyses.

## Conflict of Interest Statement

The authors declare that the research was conducted in the absence of any commercial or financial relationships that could be construed as a potential conflict of interest.

## Supplementary Material

Figure S1 **Peripheral blood leukocyte profiles in heart transplant recipients compared to healthy individuals**. Percentages of CD3+, CD20+, and CD56+ in circulation showed a significantly increased number of CD3+ T cells during rejection (****p* < 0.001).

Figure S2 **Control tissue stainings**. Tonsil and liver tissue: positive controls for CD68, CD80, and CD163 immunohistochemical stainings **(A)** tonsil and spleen: positive controls for CD14+CD16 immunofluorescence stainings **(B)**. The replacement of the secondary antibody by PBS was used as negative control.
