# Supplementary figures and images for: CD16+ Monocytes and Skewed Macrophage Polarization toward M2 Type Hallmark Heart Transplant Acute Cellular Rejection
Source: Front Immunol. 2017 Mar 24;8:346. doi: 10.3389/fimmu.2017.00346 (PMC5364145; doi:10.3389/fimmu.2017.00346)

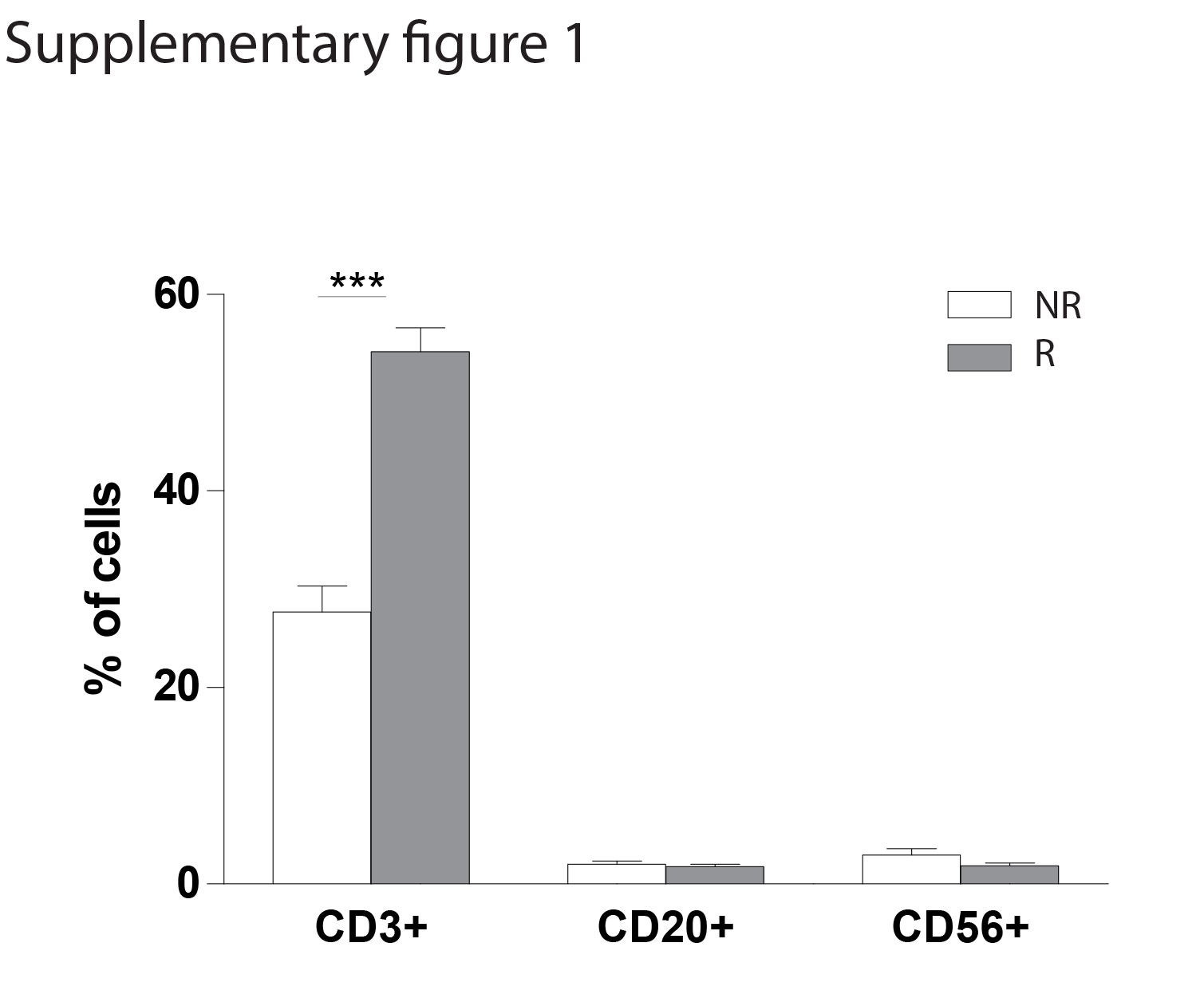

Supplement: Figure S1 — Peripheral blood leukocyte profiles in heart transplant recipients compared to healthy individuals. Percentages of CD3+, CD20+, and CD56+ in circulation showed a significantly increased number of CD3+ T cells during rejection (***p < 0.001). [file image_1.tif]

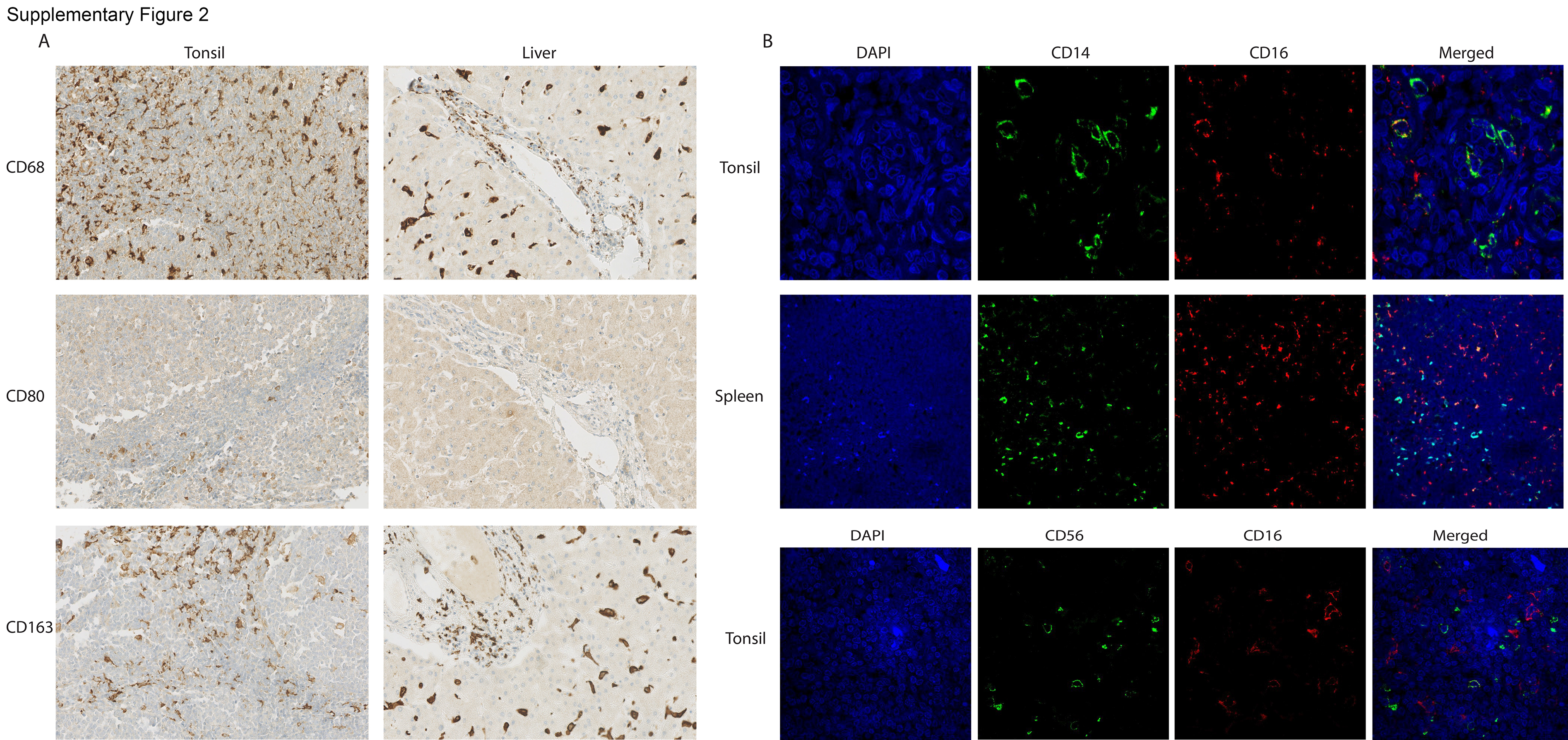

Supplement: Figure S2 — Control tissue stainings. Tonsil and liver tissue: positive controls for CD68, CD80, and CD163 immunohistochemical stainings (A) tonsil and spleen: positive controls for CD14+CD16 immunofluorescence stainings (B). The replacement of the secondary antibody by PBS was used as negative control. [file image_2.jpeg]
